# The Role of Serum Procalcitonin, Interleukin-6, and Fibrinogen Levels in Differential Diagnosis of Diabetic Foot Ulcer Infection

**DOI:** 10.1155/2018/7104352

**Published:** 2018-02-21

**Authors:** Pınar Korkmaz, Havva Koçak, Kevser Onbaşı, Polat Biçici, Ahmet Özmen, Cemile Uyar, Duru Mıstanoğlu Özatağ

**Affiliations:** ^1^Department of Clinical Microbiology and Infectious Diseases, Dumlupınar University Faculty of Medicine, 43020 Kutahya, Turkey; ^2^Department of Biochemistry, Dumlupınar University Faculty of Medicine, Kutahya, Turkey; ^3^Department of Endocrinology and Metabolism, Dumlupınar University Faculty of Medicine, Kutahya, Turkey; ^4^Department of Plastic Surgery, Kütahya Dumlupınar Training and Research Hospital, Kutahya, Turkey; ^5^Department of Clinical Microbiology and Infectious Diseases, Kütahya Dumlupınar Training and Research Hospital, Kutahya, Turkey

## Abstract

**Aims:**

We aimed to evaluate the roles of interleukin-6 (IL-6), PCT, and fibrinogen levels in the differential diagnosis of the patients with infected diabetic foot ulcer (IDFU) and noninfected diabetic foot ulcer (NIDFU) and to compare those with C-reactive protein (CRP), white blood cell (WBC), and erythrocyte sedimentation rate (ESR).

**Methods:**

Patients over 18 years with a diagnosis of type 2 diabetes mellitus and DFU who were followed up in our hospital between 1 January 2016 and 1 January 2017 were included in the study. In addition to this patient group, patients with diabetes but without DFU were determined as the control group.

**Results:**

Thirty-eight patients with IDFU, 38 patients with NIDFU, and 43 patients as the control group were included in the study. Fifty-six point three percent of the patients who participated in the study were males, and the mean age was 61.07 ± 11.04 years. WBC, ESR, CRP, IL-6, and fibrinogen levels of the cases with IDFU were determined to be significantly higher compared to the cases in NIDFU (*p* < 0.01). The area under the ROC curve (AUROC) value was highest for CRP (0.998; *p* < 0.001), and the best cut-off value for CRP was 28 m/L. The best cut-off values for fibrinogen, IL-6, ESR, and WBC were 480 mg/dL, 105.8 pg/mL, 31 mm/h, and 11.6 (103 *μ*/L), respectively.

**Conclusion:**

Serum PCT levels were not found to be effective in the discrimination of IDFU and NIDFU. Serum IL-6 and fibrinogen levels seem to be two promising inflammatory markers in the discrimination of IDFU.

## 1. Introduction

Foot infection in diabetic patients is a gradually increasing problem, and it can cause severe sequelae [1]. Infected diabetic foot ulcer (IDFU) usually develops based on the presence of skin ulceration after peripheral neuropathy or trauma. The wound is colonized by many microorganisms, and they may penetrate down to the deeper tissues and bone in consequence of the spread of infection. In cases of a progression of infection, the hospitalization of the patients, surgical resection, and amputation may be required [1]. Unfortunately, the life quality of patients undergoing lower extremity amputation is quite poor, and the five-year mortality is similar to that of some of the most mortal cancer types [2].

In a patient with a diabetic foot wound, first, the presence of infection should be assessed, and if present, the severity of the infection should be classified [3]. The classification systems of the Infectious Diseases Society of America (IDSA) and the International Working Group on the Diabetic Foot (IWGDF) are used to determine the severity of infection [1]. In the studies performed, the classification schemes used to detect the infection were found to be effective for prognosis and for the need for amputation in patients with diabetic foot ulcers [4–6].

An IDFU diagnosis should not be based on microbiological findings; clinical findings should also be used in the diagnosis [1, 7]. Since infection may rapidly deteriorate the patient's condition [2], it is necessary to diagnose IDFU rapidly [8]. However, always, it is not easy to diagnose IDFU [9]. Despite the presence of severe diabetic infection, an elevation in body temperature and leukocyte levels and in the erythrocyte sedimentation rate (ESR) may not be observed [1].

Procalcitonin (PCT) is the protein precursor of calcitonin, synthesized and released by C-cells in the thyroid gland. It is suggested that PCT production after inflammation is performed by the liver and peripheral blood mononuclear cells and is modulated by lipopolysaccharides and sepsis-related cytokines. It is also reported that PCT is a more accurate marker for a differential diagnosis of bacterial infections compared to C-reactive protein (CRP) [10]. Some studies have shown that serum PCT levels might play a role in the differential diagnosis of IDFU [11–13]. But, in another study, the role of serum PCT levels in the treatment and follow-up of infected ulcers was primarily evaluated, and then, it was reported that it had no role in the discrimination of diabetic ulcers with mild to moderate infection and severe infection [14].

Interleukin-6 (IL-6) is one of the proinflammatory cytokines that can be detected in serum in the early stages of infection. It plays a critical role, especially in the induction of CRP and fibrinogen synthesis in the liver during the course of bacterial infection. Therefore, it was suggested that this cytokine could increase earlier than CRP during bacterial infection and that it could enable an earlier diagnosis [15, 16]. There is a limited number of studies evaluating the role of serum IL-6 levels in diabetic ulcers [14, 17]. Fibrinogen and fibrin play important roles in blood clotting, fibrinolysis, cellular and matrix interactions, inflammation, wound healing, and neoplasia [18]. It was reported that serum fibrinogen levels were increased as an acute-phase protein in diabetic ulcers [17, 19].

Since there is a limited number of studies related to the use of serum IL-6, PCT, and fibrinogen levels in the diagnosis of IDFU and the results obtained are also contradictory, more advanced studies are needed on this subject. In this study, we also aimed to evaluate the roles of serum IL-6, PCT, and fibrinogen levels in the differential diagnosis both of patients with IDFU and of those with noninfected diabetic foot ulcers (NIDFU) and to compare those with other commonly used inflammatory markers like CRP, white blood cell (WBC), and ESR.

## 2. Materials and Methods

Patients over 18 years of age with a diagnosis of type 2 diabetes mellitus and diabetic foot ulcer and who were followed-up in infectious disease, internal medicine, and plastic reconstructive surgery polyclinics and clinics of our hospital between 1 January 2016 and 1 January 2017 were included in the study.

In addition to this patient group, patients with diabetes but without DFU were determined as the control group. The study was approved by the local ethics committee, and each patient was included in the study after obtaining written consent and then was informed about the study.

Patients were assessed regarding IDFU by a team including infectious disease specialists, internal medicine specialists, and plastic reconstructive surgeons. The presence of purulent discharge or two or more findings of inflammation (erythema, local warmth, local tenderness, pain, and induration) in diabetic ulcer were considered to be evidence of infection. Discrimination of IDFU and NIDFU was performed according to Infectious Diseases Society of America guidelines [19]. The patients followed up with the diagnosis of type 2 diabetes mellitus and who had no diabetic foot ulcer were determined to be the control group.

The following patients were not included in the study: the patients with other systemic or localized infectious diseases like sepsis, urinary system infection, pneumonia, and meningitis; the patients with a history of surgery within the last 6 weeks; the patients with hematological or solid malignancies; the patients with systemic inflammatory diseases like inflammatory bowel disease; the patients with rheumatoid arthritis or other rheumatic diseases; and the patients receiving ongoing immunosuppressive treatment and who received efficacious antibiotherapy earlier.

Demographic data, duration of diabetes, drugs used related to diabetes, concomitant diseases, depth of wound (superficial or deep), localization of wound (toe, metatarsal, or midfoot/heel), presence of purulent discharge, a positive probe-to-bone test, history of antibiotic use, and presence of fever were noted during admittance. Culture specimens for microbiological analysis were taken with deep tissue sampling. Osteomyelitis was assessed with the probe-to-bone test and plain-radiography [20]. Magnetic resonance imaging (MRI) was performed in patients requiring imaging examination. Blood samples were taken after 8–10 hours of overnight fasting, and complete blood count, ESR, HbA1c, fasting blood glucose, CRP, PCT, IL-6 and fibrinogen levels were studied. Complete blood count, ESR, HbA1c, fasting blood glucose, CRP, and fibrinogen levels were studied on the same day. Blood specimens for serum PCT and IL-6 levels were centrifuged at 4000 rpm for 10 minutes after storage for 30–60 minutes. Serum samples obtained were stored at −80°C until biochemical analyses were performed. Serum interleukin-6 measurements were performed by using a DiaSource Human IL-6 Elisa kit (DIAsource ImmunoAssays S.A., Belgium). Absorbance readings were performed by using a ChemWell 2910 Automated EIA and Chemistry Analyzer (Awareness Technology Inc., Martin Hwy., Palm City, USA). Results were reported as pg/mL. Serum PCT measurements were performed by using a Cobas e411 Immunoassay Analyzer (ROCHE), the electrochemiluminescence immunoassay (ECLIA) method, and Roche Diagnostics kit. The reference intervals of serum PCT levels were 0–0.05 ng/mL. Fibrinogen measurements in plasma were performed by using a coagulometer device (ACL TOP 700) and suitable kit (HemosIL Q.F.A. Thrombin). The reference intervals of serum fibrinogen levels were 200–393 mg/dL. Serum complete blood count, ESR, HbA1c, fasting blood glucose, and CRP levels were studied in the biochemistry laboratory of our hospital. All tests were performed in a blinded manner.

NCSS (Number Cruncher Statistical System) 2007 (Kaysville, Utah, USA) program was used for the statistical analysis. During the evaluation of the study data, regarding the comparisons of descriptive statistical methods (mean, standard deviation, median, frequency, ratio, and minimum and maximum) as well as quantitative data, the Mann Whitney *U* test was used for the intergroup comparisons of parameters without normal distribution. One-way ANOVA test was used for the comparisons of the groups three or more with normal distribution, and the Bonferroni test was used to determine the group causing the difference if variances were homogenous but the Games-Howell test was used if variances were not homogenous; the Kruskal Wallis test was used for the comparisons of the groups three or more without normal distribution, and the Mann Whitney *U* test was used to determine the group causing the difference.

Pearson's chi-square test and the Fisher-Freeman-Halton test were used for the comparison of qualitative data. Diagnostic screening tests (sensitivity, specificity, positive predictive value, and negative predictive value) and ROC curve analysis were applied for the determination of cut-off points for parameters. Significance was evaluated at a level of *p* < 0.05.

## 3. Results

Thirty-eight patients with IDFU, 38 patients with NIDFU, and 43 patients as the control group were included in the study. Fifty-six point three percent of the patients (*n* = 67) who participated in the study were males, and the mean age was determined to be 61.07 ± 11.04 years. Demographic data of the patients who participated in the study are shown in Table 1. Wound characteristics in the groups with IDFU and NIDFU are shown in Table 2. A positive probe-to-bone test was observed in a total of 9 cases, and osteomyelitis was determined in 4 of these cases with MRI. We detected the characteristic findings of diabetic foot osteomyelitis on MRI, decreased signal intensity of the affected bone on T1-weighted images and increased intensity on T2-weighted and postcontrast images, in these patients. Deep tissue culture was taken from 17 cases with IDFU, and microbial growth was detected in 10 (58.8%) of them. The results of microbial growth were as follows: *S. aureus* in 4 cases, *P. aeruginosa* in 2 cases, *E. cloacae* and *E. coli* in 1 case, *Streptococcus* spp. in 1 case, and *P. vulgaris* in 1 case. The results related to inflammatory markers in the groups included in the study are shown in Table 3.

WBC levels of the cases with IDFU were determined to be significantly higher compared to the cases in NIDFU (*p* < 0.01) and diabetic control groups. ESR values of the cases with IDFU were determined to be significantly higher compared to the cases with NIDFU (*p* < 0.01) and diabetic control groups. ESR values of the cases with NIDFU were determined to be significantly higher compared to the cases in the diabetic control group (*p* < 0.01). Serum CRP levels of the cases with IDFU were determined to be significantly higher compared to the cases with NIDFU (*p* < 0.01) and diabetic control groups (*p* < 0.01).

Serum CRP levels of the cases with NIDFU were determined to be significantly higher compared to the cases in the diabetic control group (*p* < 0.01).

Serum IL-6 levels of the cases with IDFU were determined to be significantly higher compared to the cases with NIDFU (*p* < 0.01) and diabetic control groups (*p* < 0.01). Serum IL-6 levels of the cases with NIDFU were determined to be significantly higher compared to the cases in the diabetic control group (*p* < 0.01). No statistically significant difference was determined between serum PCT measurements of the cases with IDFU compared to the cases with NIDFU (*p* > 0.05) and the cases in the diabetic control group (*p* > 0.05). Serum fibrinogen levels of the cases with IDFU were determined to be significantly higher compared to the cases with NIDFU (*p* < 0.01) and diabetic control groups (*p* < 0.01). Serum fibrinogen levels of the cases with NIDFU were determined to be significantly higher compared to the cases in the diabetic control group (*p* < 0.01).

**Figure 1:**
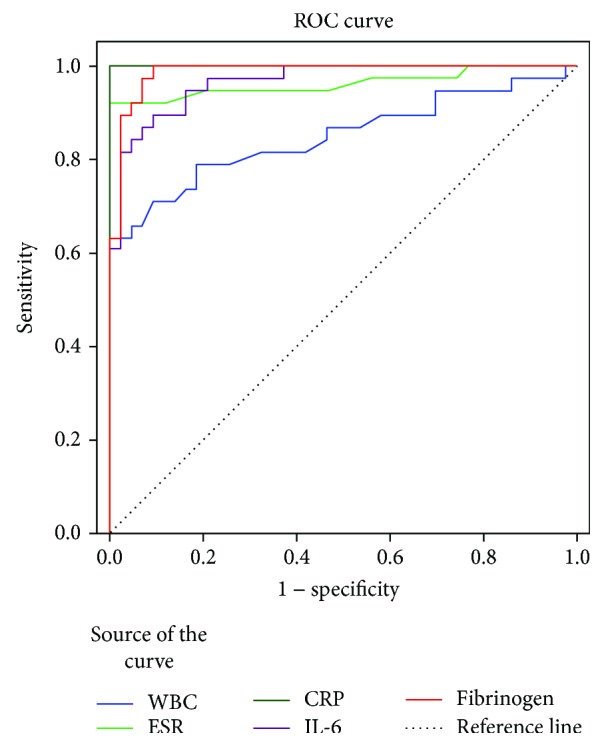
Receiver operating characteristic curves of inflammatory markers.

The area under the ROC curve (AUROC) was measured to estimate the presence of bacterial infection in the cases with diabetic ulcer (Figure 1). AUROC value was highest for CRP (0.998; *p* < 0.001), followed by ESR (0.962; *p* < 0.001), fibrinogen (0.941; *p* < 0.001), IL-6 (0.904; *p* < 0.001) and WBC (0.849; *p* < 0.001), respectively. The best cut-off values for CRP, fibrinogen, IL-6, ESR, and WBC were 28 mg/L, 480 mg/dL, 105.8 pg/mL, 31 mm/h, and 11.6 (103 *μ*/L), respectively. Maximum sensitivity, specificity, and positive and negative predictive values are shown in Table 4.

## 4. Discussion

The role of various inflammatory markers like WBC, ESR, CRP, PCT, IL-6, and fibrinogen in the discrimination of IDFU was evaluated in this study. It was shown that all inflammatory markers evaluated in our study except PCT had a role in the discrimination of IDFU. Contrary to our study, in the study performed by Uzun et al. [12], the highest discriminatory power was defined for PCT in the diagnosis of IDFU (AUROC: 0.859). In another study performed by Jonaidi Jafari et al. [9] who evaluated the role of serum PCT levels in the discrimination of IDFU and NIDFU, sensitivity and specificity were determined to be 70% and 74%, respectively, for 0.21 ng/mL value of PCT. However, in the same study, the marker with the highest discriminatory power for IDFU and NIDFU was ESR and it was followed by CRP, PCT, and WBC. The authors state that serum PCT levels may have a role in the discrimination of IDFU in the case of the combination of markers like ESR and CRP [9]. Similarly, in another study performed by Massara et al. [13], the authors stated that the highest sensitivity and specificity in the discrimination of IDFU and NIDFU could be provided with a combination of at least two markers (CRP and PCT or ESR and PCT). Also in the study performed by Jeandrot et al. [11] evaluating the role of serum CRP and PCT levels in the discrimination of mildly infected and noninfected diabetic foot ulcer, the highest AUC value (AUROC: 0.947) was obtained with the combination of CRP and PCT.

In the majority of these studies evaluating the role of serum PCT levels, the patients not receiving antibiotic 6 months before admission were included in the study. When considering the natural history of IDFU in clinical practice, this is not a frequently encountered condition. In a review, the role of serum PCT levels in the discrimination of IDFU was evaluated and it was stated that the studies were heterogeneous and the patients receiving an antibiotic within the last 6 months were excluded in many of them. They also stated that serum PCT levels might have a potential role in the discrimination of IDFU but it could not discriminate severe infection from less severe infection [21]. Also in another review evaluating IDFU, it was stated that in the absence of systemic manifestations of localized infection, serum PCT levels could not discriminate acute infection from acute ischemia or noninfectious conditions or osteomyelitis from soft tissue infections [22]. Serum PCT levels have some limitations such as the following: they cannot be studied in the laboratory of many hospitals and they are expensive markers, able to show change according to age, pathogen, and type of infection [9]. Further studies are required for routine use of serum PCT levels in IDFU diagnosis.

As far as the literature can be reviewed so far, there are only two studies related to the use of serum IL-6 levels in IDFU diagnosis. The first one was a study including also type 1 diabetes patients; it was determined in this study that serum IL-6 levels were effective in ulcer classification according to Texas classification but it was not an independent variable for the determination of infection severity [17].

The second one was a study including only the patients with IDFU; it was determined in this study that serum IL-6 levels were increased in correlation with CRP and the other inflammatory markers and serum IL-6 levels were decreased in the patients recovered with antibiotic treatment. However, this study includes only the patients followed up with the diagnosis of IDFU, and since there is no control group, it is not possible to compare baseline serum IL-6 levels of the IDFU group and the NIDFU group [14]. As far as the literature could be evaluated, it was shown for the first time in our study that serum IL-6 levels were effective for the discrimination of infected and noninfected ulcer in a study including only type 2 diabetes patients. While this shows us that serum IL-6 levels might have a role in the diagnosis of IDFU, since the number of studies related to serum IL-6 levels is extremely limited, further studies are required.

In the study performed by Rattan et al. [23], it was reported that serum fibrinogen levels were elevated in the patients with diabetic ulcer compared to the patients without diabetic ulcer. As far as the literature related to the use of serum fibrinogen levels in IDFU could be evaluated, only two studies were found. The first one of these was the study performed by Weigelt et al. [17], and it was shown that there was no significant difference between the patients with and without diabetic ulcer regarding serum fibrinogen levels. The second one of these was the study performed by Li et al. [19], and serum fibrinogen levels were found to be associated with the severity of diabetic foot ulcer and undergoing amputation. In our study, serum fibrinogen levels were found to be effective for discriminating infected diabetic foot ulceration from uninfected diabetic foot ulceration.

CRP is an acute phase reactant whose levels elevate during inflammatory processes occurring in the body; elevated serum CRP levels can also be detected in the conditions not caused by bacterial infection [12]. In a study performed, elevated serum CRP levels were determined in diabetic patients compared to nondiabetic patients and again in the patients with DFU compared to the patients without DFU. However, in this study, serum CRP levels were not found to be statistically significant especially in the discrimination of IDFU and NIDFU [12]. On the contrary, in our study, serum CRP level is the inflammatory marker which has the highest discriminatory power in the discrimination of IDFU and NIDFU. In harmony with our study, serum CRP level was determined to be the inflammatory marker with the highest discriminatory power in the discrimination of mildly IDFU and NIDFU [11]. In another study indicating that serum CRP levels were more effective than the other inflammatory markers, 123 IDFUs were evaluated and the roles of serum PCT and CRP levels in IDFU were evaluated and only serum CRP levels were found to be effective in grading the severity of the infection [24].

There are some limitations in our study; since anaerobic culture was not accessible in our hospital, anaerobic pathogens were not studied in diabetic foot ulcers. Also, the diagnosis of osteomyelitis in our study was based on imaging reports rather than bone biopsy, which is a more definite diagnostic method.

As a result, serum CRP, ESR, IL-6, fibrinogen, and WBC levels were determined to be useful parameters in the diagnosis of IDFU in our study. Serum PCT levels were not found to be effective in the discrimination of IDFU and NIDFU. Serum IL-6 and fibrinogen levels seem to be two promising inflammatory markers in the discrimination of IDFU. The efficiency of serum IL-6 levels for the discrimination of infected and noninfected ulcer in infections of ulcers associated with type 2 diabetes was shown for the first time in our study. Since serum IL-6 levels have been used in a limited number of studies, further studies are required in order to understand its role in the diagnosis of IDFU. Since especially fibrinogen can be reached easily, test results can be obtained rapidly and it is cheap; it seems to be a useful inflammatory marker in the diagnosis of IDFU. As the studies related to the role of serum fibrinogen levels in IDFU increase, the cut-off point can be determined and it may play a role in the diagnosis of IDFU together with the other inflammatory markers.

## Figures and Tables

**Table 1:** Demographic characteristics of the patients.

Characteristics		Total	Diabetic groups			*p*
			DFI (*n* = 38)	NDFI (*n* = 38)	Control (*n* = 43)	
Age (year)	Min–max (median)	29–84 (61)	40–84 (62.5)	39–81 (63)	29–80 (59)	
	Mean ± SD	61.07 ± 11.04	62.97 ± 11.47	62.24 ± 10.93	58.35 ± 10.46	0.124

Gender, *n* (%)	Male	67 (56.3)	26 (68.4)	25 (65.8)	16 (37.2)	
	Female	52 (43.7)	12 (31.6)	13 (34.2)	27 (62.8)	**0.007**

Duration of diabetes (year)	Min–max (median)	1–35 (10)	2–35 (14)	1–32 (15)	1–21 (6)	
	Mean ± SD	11.60 ± 7.73	14.11 ± 7.65	13.47 ± 8.30	7.72 ± 5.61	**0.001**

Use of insulin, *n* (%)	Absent	52 (43.7)	9 (23.7)	10 (26.3)	33 (76.7)	
	Present	67 (56.3)	29 (76.3)	28 (73.7)	10 (23.3)	**0.001**

Use of oral antidiabetic, *n* (%)	Absent	64 (53.8)	29 (76.3)	25 (65.8)	10 (23.3)	
	Present	55 (46.2)	9 (23.7)	13 (34.2)	33 (76.7)	**0.001**

Not receiving antidiabetic treatment, *n* (%)	No	103 (86.6)	28 (73.7)	35 (92.1)	40 (93.0)	
	Yes	16 (13.4)	10 (26.3)	3 (7.9)	3 (7.0)	**0.019**

Hypertension, *n* (%)	Absent	58 (48.7)	14 (36.8)	24 (63.2)	20 (46.5)	
	Present	61 (51.3)	24 (63.2)	14 (36.8)	23 (53.5)	0.067

Cerebrovascular accident, *n* (%)	Absent	115 (96.6)	37 (97.4)	36 (94.7)	42 (97.7)	
	Present	4 (3.4)	1 (2.6)	2 (5.3)	1 (2.3)	0.836

Peripheral vascular disease, *n* (%)	Absent	105 (88.2)	27 (71.1)	35 (92.1)	43 (100.0)	
	Present	14 (11.8)	11 (28.9)	3 (7.9)	0 (0.0)	**0.001**

Chronic obstructive pulmonary disease, *n* (%)	Absent	114 (95.8)	35 (92.1)	36 (94.7)	43 (100.0)	
	Present	5 (4.2)	3 (7.9)	2 (5.3)	0 (0.0)	0.187

Chronic renal failure, *n* (%)	Absent	111 (93.3)	34 (89.5)	36 (94.7)	41 (95.3)	
	Present	8 (6.7)	4 (10.5)	2 (5.3)	2 (4.7)	0.656

Coronary artery disease, *n* (%)	Absent	86 (72.3)	23 (60.5)	31 (81.6)	32 (74.4)	
	Present	33 (27.7)	15 (39.5)	7 (18.4)	11 (25.6)	0.113

Fasting blood glucose	Min–max (median)	61–718 (190)	98–718 (230.5)	61–509 (195)	62–320 (141)	
	Mean ± SD	209.82 ± 111.76	253.32 ± 128.26	223.08 ± 115.23	159.65 ± 66.76	**0.001**

HbA1c	Min–max (median)	5.60–18 (9)	6.3–13.4 (9.2)	5.6–18 (9.35)	5.8–12.8 (7.2)	
	Mean ± SD	9.18 ± 2.30	9.55 ± 1.73	9.89 ± 2.68	8.23 ± 2.11	**0.002**

IDFU: infected diabetic foot ulcer; NIDFU: non-infected diabetic foot ulcer.

**Table 2:** Evaluation of wound characteristics in the groups with diabetic ulcer.

Characteristics		Total (*n* = 76)	DFI (*n* = 38)	NDFI (*n* = 38)
		*n* (%)	*n* (%)	*n* (%)
Localization of ulcer	Toe	26 (34.2)	12 (31.6)	14 (36.8)
	Metatarsal	32 (42.1)	19 (50.0)	13 (34.2)
	Midfoot/heel	18 (23.7)	7 (18.4)	11 (28.9)

Depth of ulcer	Superficial	51 (67.1)	16 (42.1)	35 (92.1)
	Deep	25 (32.9)	22 (57.9)	3 (7.9)

Secretion	No	59 (77.6)	21 (55.3)	38 (100.0)
	Yes	17 (22.4)	17 (44.7)	0 (0.0)

Positive probe-to-bone test	No	67 (88.2)	29 (76.3)	38 (100.0)
	Yes	9 (11.8)	9 (23.7)	0 (0.0)

History of antibiotic use	No	58 (76.3)	20 (52.6)	38 (100.0)
	Yes	18 (23.7)	18 (47.4)	0 (0.0)

Fever	No	62 (81.6)	24 (63.2)	38 (100.0)
	Yes	14 (18.4)	14 (36.8)	0 (0.0)

IDFU: infected diabetic foot ulcer; NIDFU: noninfected diabetic foot ulcer.

**Table 3:** Inflammatory markers in infected diabetic foot ulcer (DFI), noninfected diabetic foot ulcer (NDFI), and control groups.

	Total	^1^DFI (*n* = 38)	^2^NDFI (*n* = 38)	^3^Control (*n* = 43)	*p*	^1-2^ *p*	^1–3^ *p*	^2-3^ *p*
*WBC*								
Min–max (median)	4–44.3 (9.6)	5–44.3 (13.7)	4–15.3 (9)	4.4–12.8 (8.4)				
Mean ± SD	10.7 ± 5.5	15.2 ± 7.5	9 ± 2.3	8.4 ± 2	**0.001**	**0.001**	**0.001**	0.237
*ESR*								
Min–max (median)	2–109 (24)	6–109 (56.5)	2–64 (26)	2–30 (11)				
Mean ± SD	31.66 ± 25.89	58.34 ± 24.68	27.21 ± 16.44	12.02 ± 7.23	**0.001**	**0.001**	**0.001**	**0.001**
*CRP*								
Min–max (median)	0.5–309 (8.7)	28–309 (195)	0.5–44 (9.1)	0.5–9 (2.7)				
Mean ± SD	62.43 ± 92.35	181.17 ± 76.36	10.52 ± 7.53	3.37 ± 2.6	**0.001**	**0.001**	**0.001**	**0.001**
*IL-6*								
Min–max (median)	4.4–1717.9 (40.8)	30.7–1717.9 (191.4)	6.6–576.7 (35.35)	4.4–152.2 (24.4)				
Mean ± SD	116.22 ± 221.98	275.12 ± 331.44	55.81 ± 90.21	29.18 ± 24.35	**0.001**	**0.001**	**0.001**	**0.004**
*PCT*								
Min–max (median)	0.02–10.30 (0.14)	0.02–10.3 (0.15)	0.02–0.41 (0.12)	0.04–0.64 (0.17)				
Mean ± SD	0.31 ± 0.98	0.6 ± 1.7	0.15 ± 0.09	0.19 ± 0.14	0.468	0.261	0.708	0.341
*Fibrinogen*								
Min–max (median)	105–1182 (357)	387–1182 (627)	105–840 (350.5)	200–568(293)				
Mean ± SD	436.54 ± 204.73	663.21 ± 185.24	360.34 ± 119.6	303.56 ± 71.28	**0.001**	**0.001**	**0.001**	**0.005**

IDFU: infected diabetic foot ulcer; NIDFU: noninfected diabetic foot ulcer; CRP: C-reactive protein; IL-6: interleukin-6; WBC: white blood cell; ESR: erythrocyte sedimentation rate; PCT: procalcitonin.

**Table 4:** Sensitivity, specificity, negative predictive value, and positive predictive value of inflammatory marker.

	Cut-off	Sensitivity	Specificity	Positive predictive value	Negative predictive value
CRP (mg/L)	≥28	100.00	97.37	97.40	100.00
Fibrinogen (mg/dL)	≥480	89.47	92.11	91.90	89.70
IL-6 (pg/mL)	≥105.8	76.32	94.74	93.50	80.00
ESR (mm/h)	≥42	73.68	84.21	82.40	76.20
WBC (10^3^ *μ*/L)	≥11.6	71.05	90.70	87.10	78.00

CRP: C-reactive protein; IL-6: interleukin-6; WBC: white blood cell; ESR: erythrocyte sedimentation rate; PCT: procalcitonin.
